# A framework for integrating inferred movement behavior into disease risk models

**DOI:** 10.1186/s40462-022-00331-8

**Published:** 2022-07-24

**Authors:** Eric R. Dougherty, Dana P. Seidel, Jason K. Blackburn, Wendy C. Turner, Wayne M. Getz

**Affiliations:** 1 Department of Environmental Science, Policy, and Management, University of California Berkeley, Berkeley, CA USA; 2 Spatial Epidemiology and Ecology Research Laboratory, Department of Geography, University of Florida, Gainesville, FL USA; 3 Emerging Pathogens Institute, University of Florida, Gainesville, FL USA; 4 U.S. Geological Survey, Wisconsin Cooperative Wildlife Research Unit, Department of Forest and Wildlife Ecology, University of Wisconsin-Madison, Madison, WI USA; 5 School of Mathematical Sciences, University of KwaZulu-Natal, Durban, South Africa

**Keywords:** Resource selection, Step selection function (SSF ), Ecological niche models (ENM ), Animal movement, Animal behavior, Disease transmission, Epizootic, Anthrax

## Abstract

Movement behavior is an important contributor to habitat selection and its incorporation in disease risk models has been somewhat neglected. The habitat preferences of host individuals affect their probability of exposure to pathogens. If preference behavior can be incorporated in ecological niche models (ENMs) when data on pathogen distributions are available, then variation in such behavior may dramatically impact exposure risk. Here we use data from the anthrax endemic system of Etosha National Park, Namibia, to demonstrate how integrating inferred movement behavior alters the construction of disease risk maps. We used a Maximum Entropy (MaxEnt) model that associated soil, bioclimatic, and vegetation variables with the best available pathogen presence data collected at anthrax carcass sites to map areas of most likely *Bacillus anthracis* (the causative bacterium of anthrax) persistence. We then used a hidden Markov model (HMM) to distinguish foraging and non-foraging behavioral states along the movement tracks of nine zebra (*Equus quagga*) during the 2009 and 2010 anthrax seasons. The resulting tracks, decomposed on the basis of the inferred behavioral state, formed the basis of step-selection functions (SSFs) that used the MaxEnt output as a potential predictor variable. Our analyses revealed different risks of exposure during different zebra behavioral states, which were obscured when the full movement tracks were analyzed without consideration of the underlying behavioral states of individuals. Pathogen (or vector) distribution models may be misleading with regard to the actual risk faced by host animal populations when specific behavioral states are not explicitly accounted for in selection analyses. To more accurately evaluate exposure risk, especially in the case of environmentally transmitted pathogens, selection functions could be built for each identified behavioral state and then used to assess the comparative exposure risk across relevant states. The scale of data collection and analysis, however, introduces complexities and limitations for consideration when interpreting results.

## Background

Animal space use depends on the dynamic interplay between the internal state of an individual and the heterogeneous landscape over which it moves [1]. Landscape heterogeneity is structured by the underlying eco-evolutionary dynamics [2], ranging from readily measurable features such as vegetation type or canopy cover to more elusive features, such as infection risk. Ultimately, animal movement decisions are made based on trade-offs between the benefits of satisfying physiological needs and the costs of potential encounters with competitors, predators, or pathogens [3].

With recent advancements in the technologies that track animal positions through time [4], a number of path segmentation methods have been developed to parse movement tracks into behavioral states and more clearly interpret the motivations underlying the decision to move [5, 6]. Such analytical methods offer insight into the space use patterns of animals during specific activity modes, allowing researchers to understand how resource selection differs depending on the internal state of an individual.

In an impressive meta-analysis of 859 habitat selection studies, McGarigal and colleagues [7] identified only nine studies in which multi-level analyses of different behavioral states were treated as giving rise to differential habitat selection. These studies ranged across taxa and geographical regions, from the wandering albatross (*Diomedea exulans*) in the Southern Ocean [8] to the Canada lynx (*Lynx canadensis*) in the Northern Rocky Mountains [9]. In these cases and others, the incorporation of behavioral state resulted in distinctly different conclusions regarding the space use patterns of the animals (e.g., [10–17]). Noting this important trend, others have emphasized the potential implications of ignoring behavioral state when considering habitat selection and animal space use patterns [18], particularly in the context of conservation [19, 20]. Despite this recognition, applications of behavioral analysis methods in habitat selection studies are still rare.

Notably, considerations of behavioral state have not yet permeated the literature regarding the transmission of disease, where host behavior is a fundamental element of pathogen spread. This is particularly the case for pathogens transmitted via environmental reservoirs [6, 21]. The spatial distribution of such pathogens can be readily modeled using remotely-sensed proxies of various environmental factors [22], making studies of their overlap with host animals especially fruitful. Two prior studies of such overlap were carried out based on seasonal habitat preference using a resource selection function (RSF) framework [23, 24]; while informative, these studies did not include avoidance behavior at the much finer step selection level [25, 26], as we present here.

The importance of particular behavioral states inferred from telemetry data has received limited attention, but the implications of excluding this information could be significant [27]. For example, habitat selection studies may offer insight into the evolutionary struggle between host and pathogen. If a pathogen is able to persist in areas that are favored by a host species, they have the potential to form an ecological trap [28], thereby increasing infections in the population due to host habitat preferences.

On the other hand, host animals often adjust their habitat selection when environments fluctuate or seasons change [29], with the perhaps inadvertent consequence of avoiding the highest exposure risk areas (e.g., [30]). An analysis performed on a full movement track may result in dramatically different estimates of selection coefficients than an analysis of periods when the host behaves in a way that may promote transmission (e.g., during feeding, [31–33]). This more nuanced approach may indicate that the pathogen is more likely (e.g., if the selection coefficient for the habitat type that harbors the pathogen is higher than in the alternative analysis) or less likely (e.g., if the selection coefficient is lower) to be transmitted than otherwise predicted when behavior is ignored. No matter the direction of the difference, the exclusion of behavioral information is likely to lead to an inaccurate interpretation of pathogen exposure risk. Similarly, the direct consideration of alternative behavioral states could illuminate important differences in the ways individuals mitigate risk at particular times, with implications for wildlife management.

The unique nature of environmentally-transmitted pathogens makes them ideal for demonstrating the importance of incorporating behavior in habitat selection studies. Here we use a set of movement tracks collected from a system harboring one such pathogen, *Bacillus anthracis*, the causative agent of anthrax. Based on extensive research suggesting that the primary route of anthrax infections in ungulate species is through ingestion [31, 34, 35], we deduce that considerations of foraging behavior are important for judging the risk of infection [33]. Thus, we compare habitat selection models constructed using points assigned to the “foraging” versus the “directed” movement states; where the risk of pathogen exposure is likely significantly lower in the latter.

Fundamentally, our goal is to lay out a framework for considering both landscape-level disease risk and individual-level behavioral state and explore how the dynamics between the two may give rise to the observed movement patterns of animals. We do this by directly incorporating maps of predicted pathogen persistence in habitat selection models, enabling direct comparisons of resource use patterns and exposure risk across behavioral states. Ultimately, we demonstrate that the selection patterns that emerge when we explicitly consider behavioral state are markedly different from an analysis that considers entire trajectories without accounting for different activity modes.

## Materials and methods

### Study site and disease system

For the sake of completeness, we provide a brief summary of the study site and disease system for which more details can be found elsewhere [22, 29]. Etosha National Park (ENP) is a fenced reserve (22,270 km$^2$) located in the semi-arid savannah of northern Namibia. In terms of seasons, ENP is relatively cool and dry May–August, hot and semi-dry September-December, and hot and wet January–April. The average annual rainfall in the area associated with the data used in this study is 358 ± 127 mm. The vegetation in our study area is primarily grassland interspersed with shrub/tree veld populated primarily with mopane (*Colophospermum mopane*) trees and bordering a large salt pan.

Anthrax is endemic in ENP, and plains zebra are the most common host species, constituting more than 50% of cases. Zebra anthrax mortalities peak in the late wet season (March–April) where case numbers are positively correlated with annual rainfall [31, 36]. Anthrax environmental reservoirs, known as locally infectious zones (LIZs, [37]) are associated with positive carcass sites from current and prior years. Transmission is greatly enhanced when zebra graze at these LIZs [32, 33]. Hence, a recent history (i.e., going back 1–5 years) of a relatively high number of anthrax carcasses in a particular area increases the risks of exposure in that area compared with areas that had fewer carcasses over time.

### Movement data preparation

Analyses were conducted on GPS tracks collected from zebra in Etosha National Park in Namibia in 2009 and 2010. Step-selection functions were developed for the nine zebra (*Equus quagga*) for which GPS points were recorded during the anthrax seasons of those years (defined as the five-month period between February 1 and June 30; [31, 38]). This temporal criterion resulted in a dataset consisting of five tracks recorded during the 2009 season and six during the 2010 season. By splitting up tracks by season, the nine zebra produced eleven separate tracks, with two individuals having long enough tracks to be represented during both seasons (Table 1). The GPS track of each zebra consisted of positional fixes collected 20 minutes apart.

**Table 1:** Summary of the eleven regularized zebra tracks for which step-selection functions were developed

Animal ID	Number of Points	Missing Points	Start Date	End Date
AG059	4824	5	2009-04-25	2009-06-30
AG061	4824	152	2009-04-25	2009-06-30
AG062	4824	646	2009-04-25	2009-06-30
AG063	4824	7	2009-04-25	2009-06-30
AG068	4824	11	2009-04-25	2009-06-30
AG063	6331	86	2010-02-01	2010-04-30
AG068	10,800	2,072	2010-02-01	2010-08-29
AG252	10,800	39	2010-02-01	2010-08-29
AG253	10,800	739	2010-02-01	2010-12-17
AG255	10,800	28	2010-02-01	2010-08-29
AG256	10,800	2	2010-02-01	2010-08-29

Note that individuals AG063 and AG068 had tracks that spanned two anthrax seasons, resulting in two separate entries here

### Anthrax risk map

A predictive layer of anthrax risk was created using an ensemble ecological niche modeling approach. Separate maps were created for the 2009 and 2010 anthrax seasons based on the presence-only data gathered from sites in Etosha National Park that contained anthrax spores at least one year after the deposition of the carcass [33]. The carcass data consisted of 40 points at sites that contained non-zero concentrations of anthrax spores (in colony-forming units per gram) during sampling one and two years following initial deposition. Of these 40 sites, 26 were associated with carcasses deposited in 2010, 4 with carcasses deposited in 2011, and 11 with carcasses deposited in 2012. Studies show that individual zebra avoid carcass sites for several months after they are created but are attracted to them during subsequent years, when these sites are still contaminated [32]. Thus, the risk of infection with anthrax in 2009 and 2010 will depend upon carcass sites from 2007-2008 and 2008-2009, respectively. It should be noted that the carcasses used to derive the anthrax risk layers do not represent an exhaustive or random record of anthrax-positive zebra carcasses in the Etosha region (fewer than 25% of carcasses from zebra that have died of anthrax are likely to have been observed [39]). Additionally, our map does not account for springbok, elephant, and wildebeest anthrax-positive carcasses. Thus, our study is more methodological and illustrative than definitive.

Others have created predictions for *B. anthracis* based on ecological niche models (ENMs; e.g., [23, 40, 41]), but due to the site-specific nature of the data used, they tend to be applicable only in the region for which they are built [42]. Despite their specificity, these models do offer insight into potential predictor variables for *B. anthracis* persistence and can inform the niche model constructed here (Table 2). Because the carcass data used in developing this particular niche modeling experiment represent sites at which anthrax spores were able to persist for multiple years, the risk map does not simply serve as a predictive map of carcasses. Rather, it relates *B. anthracis* persistence to the soil, bioclimatic, and vegetation predictors at sites previously occupied by a carcass. Given that these locations represent presence-only data, we applied Maximum Entropy methods [43] to an initial predictor variable set consisting of three general categories: soil characteristics, bioclimatic variables, and vegetation indices (see the Supplementary Materials for information on these variables).

We parameterized our MaxEnt models by first generating a set of random ‘pseudo-absence’ locations on our map—we decided on 500—in proportion to the number of carcasses observed in each of three seasons for which we had data to obtain 312, 50, and 138 pseudo-absence points respectively associated with 2010, 2011, and 2012 seasons. The predictor values were extracted for each presence and pseudo-absence point according to its deposition year. An initial MaxEnt model was run on these data and the full candidate predictor set using the implementation in the dismo package (version 1.1–4; [44]) in R (version 3.4.3; [45]). Following an investigation of the variable contributions to this full model (generated as a standard output of the maxent function), variables exhibiting covariance with another predictor were culled such that the variable in the pair with the higher contribution to the MaxEnt model was maintained and its counterpart eliminated. Finally, another MaxEnt model was run on the reduced predictor variable set.

In order to obtain an anthrax risk map for both 2009 and 2010, the MaxEnt model was projected onto the environmental predictor variable sets associated with those years (Additional file 1: Figs. S1 and S2). In 2009, this meant that the vegetation indices were calculated over the period from 2007 to 2009, and for the 2010 risk map, the vegetation indices were calculated over the period between 2008 and 2010. The continuous risk layers were directly incorporated into the step selection functions described below. For the sake of visualization, however, we followed the approach set forth in [23] and used three thresholds representing liberal, moderate, and conservative cutoffs to generate a discretized version of the risk layers. This enabled clearer delineation of the geographic range of risk, or pertinent transmission zone (PTZ), but these discrete layers were not used for habitat selection modeling.

**Table 2:** Set of potential predictor variable layers used in creating the anthrax risk map

Environmental variable (units)	Predictor name	Data source	Final Model
Soil pH x 10 in $H_2O$	pH	SoilGrids$^{*}$	X
Soil Organic Carbon Content (g/kg)	OC	SoilGrids$^{*}$	X
Soil Cation Exchange Capacity (cmolc/kg)	CEC	SoilGrids$^{*}$	X
Mean annual temperature (C$^{\circ }$)	bio1	WorldClim$^{\dagger }$	X
Annual temperature range (C$^{\circ }$)	bio7	WorldClim$^{\dagger }$	X
Annual precipitation (mm)	bio12	WorldClim$^{\dagger }$	
Precipitation of the wettest month (mm)	bio13	WorldClim$^{\dagger }$	X
Precipitation of the driest month (mm)	bio14	WorldClim$^{\dagger }$	
Mean NDVI	NDVI	Landsat 7$^{\ddagger }$	
Maximum NDVI	max_ndvi	Landsat 7$^{\ddagger }$	X
Minimum NDVI	min_ndvi	Landsat 7$^{\ddagger }$	X
Range NDVI	range_ndvi	Landsat 7$^{\ddagger }$	X

These predictors were compiled based on their use in similar ecological niche modeling efforts of *Bacillus anthracis* (see [40] and [23] for more details). Several of these variables were eliminated, however, due to collinearity with other, more important, variables in the set. An ‘X’ in the ‘Final Model’ column indicates the inclusion of that variable in the final MaxEnt model. Data sources: $^{*}$ [46]; $^{\dagger }$ [47]; $^{\ddagger }$ courtesy of the U.S. Geological Survey (https://espa.cr.usgs.gov/)

### Behavioral analysis

We used a hidden Markov model (HMM; [48–50]) to probabilistically assign one of three different behavioral states, interpreted as resting (state 1: step size $10^{0}$ to $10^{1}$, lack of directionality), foraging (state 2: step size $10^{1}$ to $10^{2}$, moderate directionality) and directional movement (state 3: step size $10^{2}$ to $10^{3}$, persistent directionality). The points that were assigned to the ‘foraging’ and ‘directed movement’ states formed the basis of the two reduced datasets used for the behaviorally-conditioned step-selection function described below. Each track was analysed separately to more accurately reflect the variability among individuals and properly parameterize the animal-specific step-length distributions used in subsequent analyses. The parameters governing the step length and turning angle distributions of the three behavioral states can be seen in Additional file 1: Tables S1–S11. In subsequent analyses, the means ($\mu$) and standard deviations ($\sigma$) in these tables are transformed into the more traditional shape and rate (i.e., the inverse of scale) parameters of the gamma distribution according to:

$$\begin{aligned} \text {shape} = \frac{\mu ^{2}}{\sigma ^{2}}, \qquad \qquad \text {rate} = \frac{\mu }{\sigma ^{2}} \end{aligned}$$

Our three-state model is not meant to capture all of the nuance of zebra behavior, but to strike a balance between flexibility and interpretability (see [51] for further discussion). The parameters defining these states provide a way for us to infer whether or not the individual had likely made a decision to leave a landscape cell defined by the 30 m raster resolution of our maps during the relocation sampling period. Specifically, if the mean step length of the behavioral state at a location was less than 42.4 meters (the diagonal distance across a 30 meter cell), then we scored the individual as unlikely to have made a concerted movement decision, and the portion of the track assigned to this ‘low-movement’ location would be omitted from the analysis. In our case, the movement state that we refer to as ‘resting’ was relegated to this category and thus, does not appear in the final results presented here.

### Step-selection function

The step-selection function (SSF) procedure implemented here follows that of Zeller et al. [16] (later used in [26] and [52]) with some minor adjustments.

Our SSF method consisted of the following steps:

1. We fitted a separate Gamma probability distribution (but see [16]) to the distribution of step lengths from each individual and set a maximum threshold of 97.5 percentile, as recommended by Zeller et al. in setting up our fitted distribution as a step length movement kernel. This threshold effectively represented the maximum movement range (i.e., radius) of individuals at the scale of each 20 minute time step (Table 3).
2. The maximum movement radius so obtained was then used to construct an ‘available area’ buffer around each ‘used’ point, thereby greatly reducing the computational costs associated with including cells that fall within the long tail of the gamma distribution. Unlike Zeller *at al.* [16], we omitted the construction of a separate 30-meter GPS error buffer around each ‘used’ point, because the resolution of the underlying predictor layers relative to the locational error of the GPS units rendered this correction moot.
3. For each continuous variable, the density function of the estimated step length distribution was applied to weight the value of each cell based on its distance from the ‘used’ point. This procedure places higher weights on areas closer to the ‘used’ point and lower weights on those farther away. Only those cells whose center falls within the buffer are considered in the calculation of the weighted mean. We then paired the weighted mean value, reflecting a summary of the ‘available’ area, with the values extracted at the ‘used’ point. These pairs of predictor value sets form the basis of the conditional logistic regression.
4. We fitted the following conditional logistic regression selection model (response variable *w*, predictors $x_1$ to $x_n$, and selection ratios $\beta _i$, $i=1,...n,$—$\beta _i<0$ indicates selection against the $i^{th}$ predictor variable)
  $$\begin{aligned} w(x_1,...,x_n) = e^{\beta _1x_1 + \cdots + \beta _n x_n } \end{aligned}$$
  [53, 54] so that we could relate the set of values $x_n$ arising from each ‘used’ area with a response value $w_T$ in the associated ‘available’ area [55–57], where we used *T* to refer to the year of the particular data set used.
5. We included individual ID as a random effect [58, 59] variable in our model alongside our fixed effects of Greenness ($$x_1$$), Wetness ($$x_2)$$, Road Density ($$x_3$$), and Anthrax Risk ($$x_4$$). We repeated this analysis separately for each of the two seasons, thereby deriving $$w_{2009}$$ and $$w_{2010}$$ as separate step selection functions (SSFs). The generation of separate SSFs for 2009 and 2010 allows us to check consistency across consecutive years.

Conventional SSF approaches overcome much of the subjectivity associated with home range delineation methods required of traditional resource selection function methods [60] by selecting a certain number of ‘available’ points for each ‘used’ point based on empirical step length and turning angle distributions [54, 61, 62]. The approach we take here offers the same benefits of directly accounting for temporal autocorrelations within movement data. Our method has an additional advantage of overcoming the approximation error associated with small samples of ‘available’ points [63]. By censusing the entire available area, one can estimate the categorical variable proportions and continuous variable distributions of predictor values associated with a given ‘used’ point, thereby conforming to a context-dependent methodology [64].

**Table 3:** Radii of the kernels (in meters) used for in producing the step-selection functions for each individual

Animal ID	Kernel Radius (All)	Kernel Radius (Foraging)	Kernel Radius (Directed)
AG059_2009	1131	667	1532
AG061_2009	739	273	1190
AG062_2009	837	240	1148
AG063_2009	985	581	1534
AG068_2009	1183	607	1595
AG063_2010	1256	626	1686
AG068_2010	1236	590	1636
AG252_2010	1012	341	1450
AG253_2010	1101	499	1702
AG255_2010	1056	324	1502
AG256_2010	1014	376	1479

Separate radii were used for the full datasets, the foraging only dataset, and the directed movement only datasets

## Results

### Anthrax risk map

**Fig. 1:**
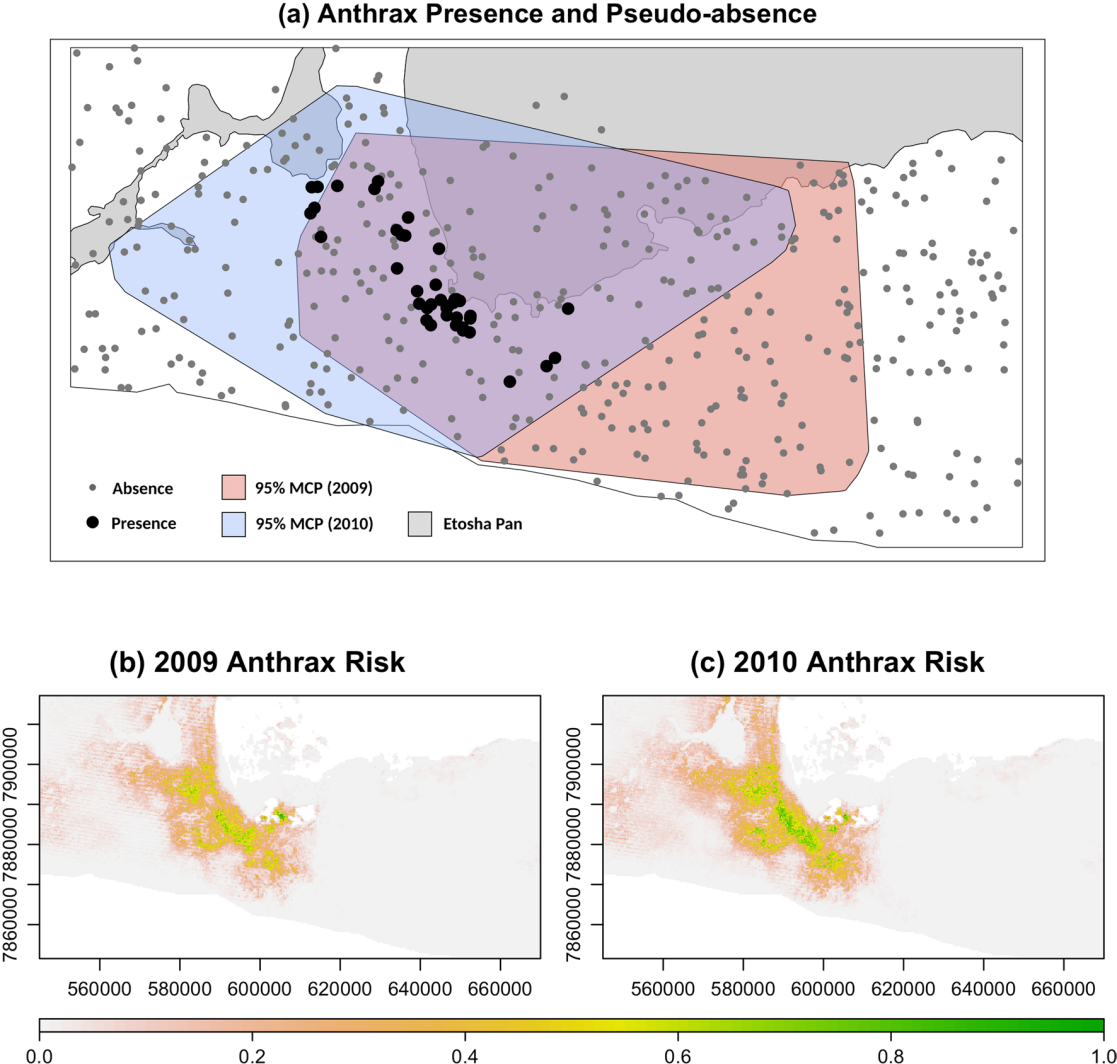
**Ecological Niche Model for Anthrax** MaxEnt derived maps of suitability for *Bacillus anthracis* persistence within the region of interest in Etosha National Park, Namibia. Panel **a** illustrates the spatial distribution of soil samples with non-zero concentrations of anthrax recorded one and two years following carcass deposition (large black dots) and background sampling points for the MaxEnt algorithm (small grey dots). The grey polygon demarcates the Etosha Pan while the red and blue polygons represent the 95% minimum convex polygon (MCP) for the zebra in the study during 2009 and 2010, respectively. Panels **b** and **c** are the predictive maps of suitability for anthrax spores in 2009 and 2010, respectively, based on the MaxEnt model created from the presence and background points in panel **a**

The MaxEnt anthrax risk maps produced for the 2009 and 2010 seasons were based on 40 presence points and 422 background points (after 78 haphazardly distributed points were removed for missing at least one of the environmental predictors; Fig. 1a). The final model incorporated a set of nine continuous predictors after the elimination of annual precipitation (bio12) and mean NDVI, which were highly correlated with precipitation of the wettest month (bio13) and maximum NDVI, respectively (see Additional file 1: Tables S12–S14 for the covariance matrices in 2010, 2011, and 2012, and Additional file 1: Table S15 for the variable contributions associated with the full model).

The variable importance table indicates that the bioclimatic and soil characteristics were larger contributors than the vegetation indices (Table 4). Mean temperature range dominated the model, contributing 73% to the final model. Soil organic carbon content was the next highest contributor at just over 11%. The vegetation measures contributed a total of only 7% to the model, despite being more temporally specific than the other measures (model coefficients and associated feature classes emerging from the MaxEnt algorithm are available in Additional file 1: Table S16).

**Fig. 2:**
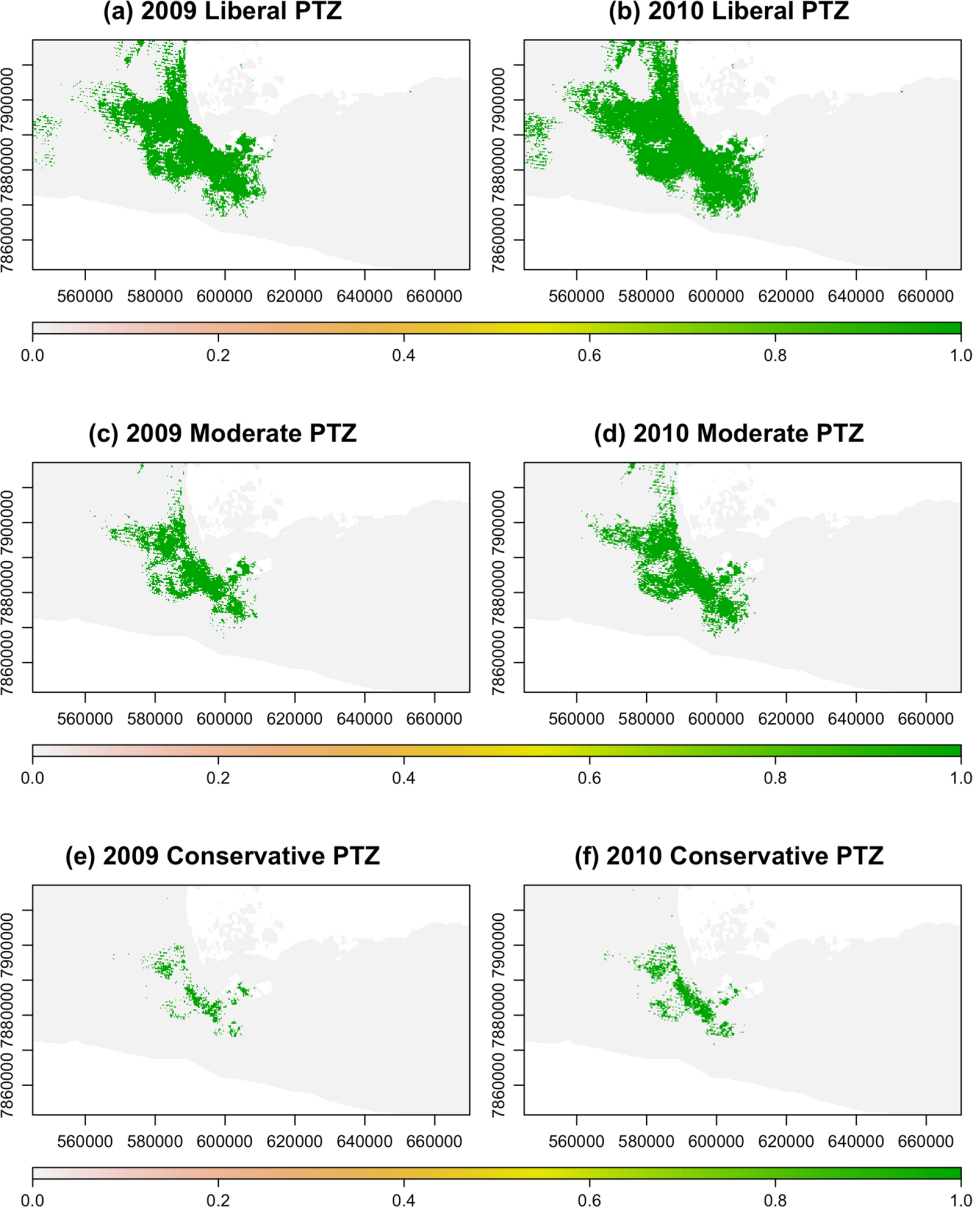
**Pertinent transmission zones (PTZs) for anthrax** Three different thresholds were used to delimit the PTZs: $>10\%$, $>25\%$, and $>50\%$ probability of suitability, corresponding to a liberal (**a**, **b**), moderate (**c**, **d**), and conservative (**e**, **f**) estimates of the area in which anthrax is likely to persist, respectively. The two columns represent the same three thresholds applied to the 2009 season (left column) and 2010 season (right column)

The final predictive maps for both 2009 and 2010 show that the greatest level of risk occurs at the southwestern edge of the Etosha pan (Fig. 1, Panels b and c). The geographical range of risk appears to be considerably larger during the 2010 season than during the 2009 season. These differences are likely driven by differences in the vegetation layers because the soil characteristic and bioclimatic variables are static between the years. To examine the qualitative differences in risk between years, the three PTZs were mapped and compared (Fig. 2). In 2009, the area defined as the PTZ, based on our most liberal definition of risk (associated with a suitability value of > 0.1), was approximately 730 km$^{2}$. The same liberal cutoff in 2010 results in a PTZ of over 943 km$^{2}$. The moderate threshold (with a suitability value of > 0.25) offers a similar impression of the disparity in PTZ size across seasons, with 2009 having a PTZ of about 344 km$^{2}$ and 2010 having one of over 463 km$^{2}$. Finally, the difference between anthrax seasons is even more pronounced when the most conservative threshold (a suitability value > 0.5) is applied, with 2009 having a PTZ of just under 77 km$^{2}$ and 2010 having a PTZ that is nearly twice as large (133 km$^{2}$). The PTZs in 2009 represent 10.4%, 4.9%, and 1.1% of the total area in the $\approx$ 7000 km$^{2}$ region of interest in Etosha National Park for the liberal, moderate, and conservative thresholds, respectively. The PTZs in 2010, however, represent 13.5%, 6.6%, and 1.9% of the total area for the same thresholds.

We note that both risk layers are characterized by a diagonal striping pattern that is an artifact of a malfunction in the scan line detector during the Landsat 7 mission [65]. Our focus in this case is to demonstrate our methods, so we used the Landsat images without applying any form of correction. Despite the limitations described, the environmental layers upon which the risk layer is built represent the best available data.

**Table 4:** Variable contribution and importance results from the final MaxEnt model of *Bacillus anthracis* persistence, built on the reduced environmental predictor set following the elimination of annual precipitation (bio12) and mean_ndvi due to covariance

Variable	Name	Percent contribution	Permutation importance
Mean temperature range	bio7	73	80
Soil Organic Carbon Content	OC	11.2	2.6
Precipitation of the wettest month	bio13	6.5	7.1
Range of NDVI	range_ndvi	4.7	2.3
Maximum NDVI	max_ndvi	2	1.6
Mean annual temperature	bio1	1.2	1.5
Soil Cation Exchange Efficiency	CEC	0.6	2.1
Soil pH	pH	0.5	0.5
Minimum NDVI	min_ndvi	0.3	2.3

### Step-selection function

To verify the efficacy of the step-selection function method we randomly generated two rasters from the largest dataset (consisting of all movement points in 2010). This allowed us to verify that no methodological artifacts, including sample size, were artificially inflating the significance of various predictors. These results, which demonstrated that zebra did not exhibit a meaningful selection of the random layers, are presented in Additional file 1: Table S17.

**Fig. 3:**
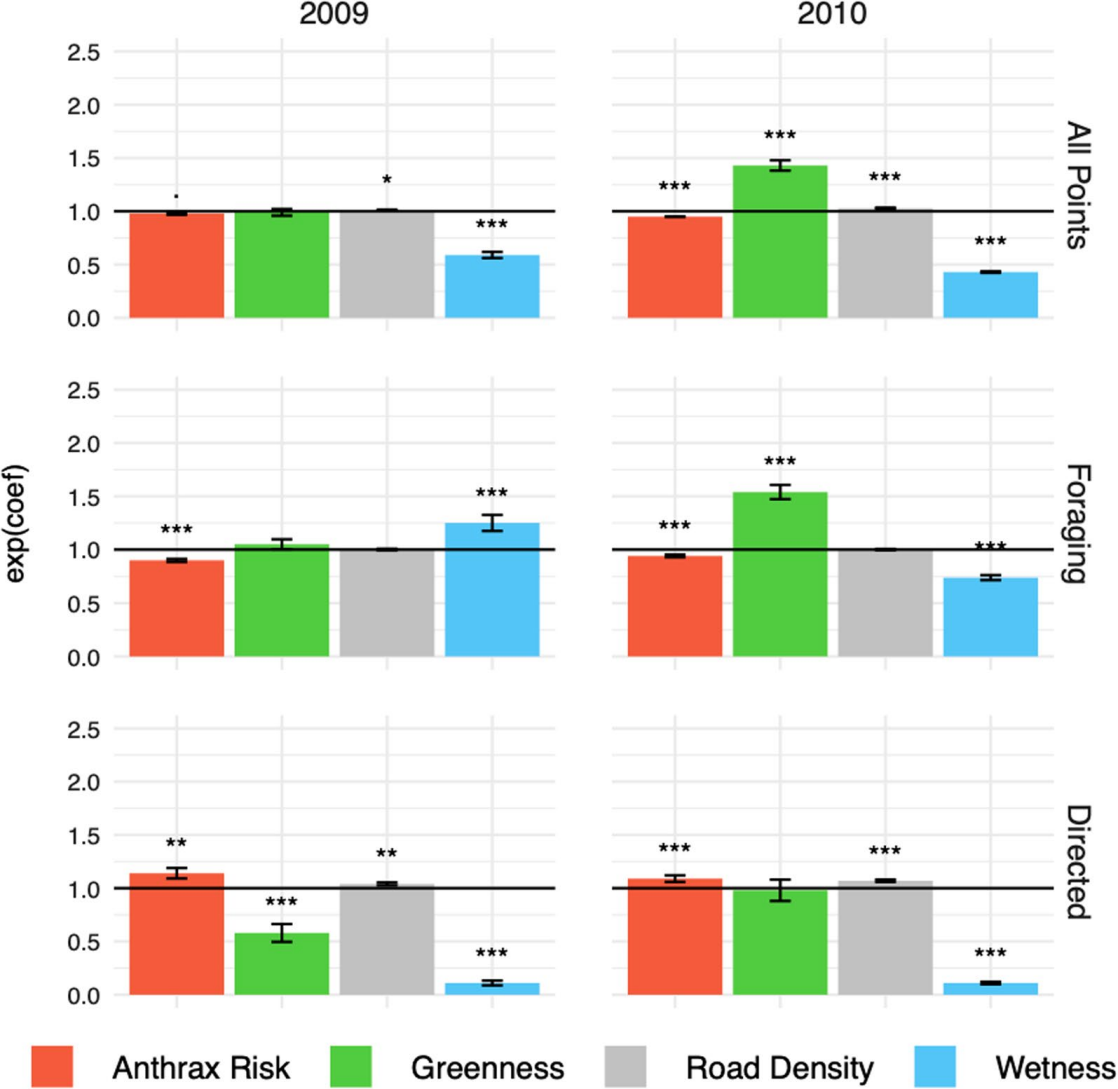
**Results of the conditional logistic mixed effects models** The bars represent the exp(coef) for each of the variables. Thus, values above one represent positive selection (preference) and those below one represent negative selection (avoidance). The first row displays the results of analyses when applied to all of the movement points (*n* = 22,949 in 2009 and *n* = 56,495 in 2010). The middle row shows results when only the foraging points (*n* = 11,733 in 2009 and *n* = 27,898 in 2010) are considered. The bottom row reflects the results when only the directed movement points (*n* = 4,381 in 2009 and*n* = 11,486 in 2010) are included. Each bar represents the normalized model coefficients (via exponentiation) with standard error bars. In addition, indicators of statistical significance are plotted above each bar, with a **.** reflecting $p < 0.1$, * signifying $p < 0.05$, ** indicating $p < 0.01$, and *** to denote factors that are significant below a threshold of $p = 0.001$

**Fig. 4:**
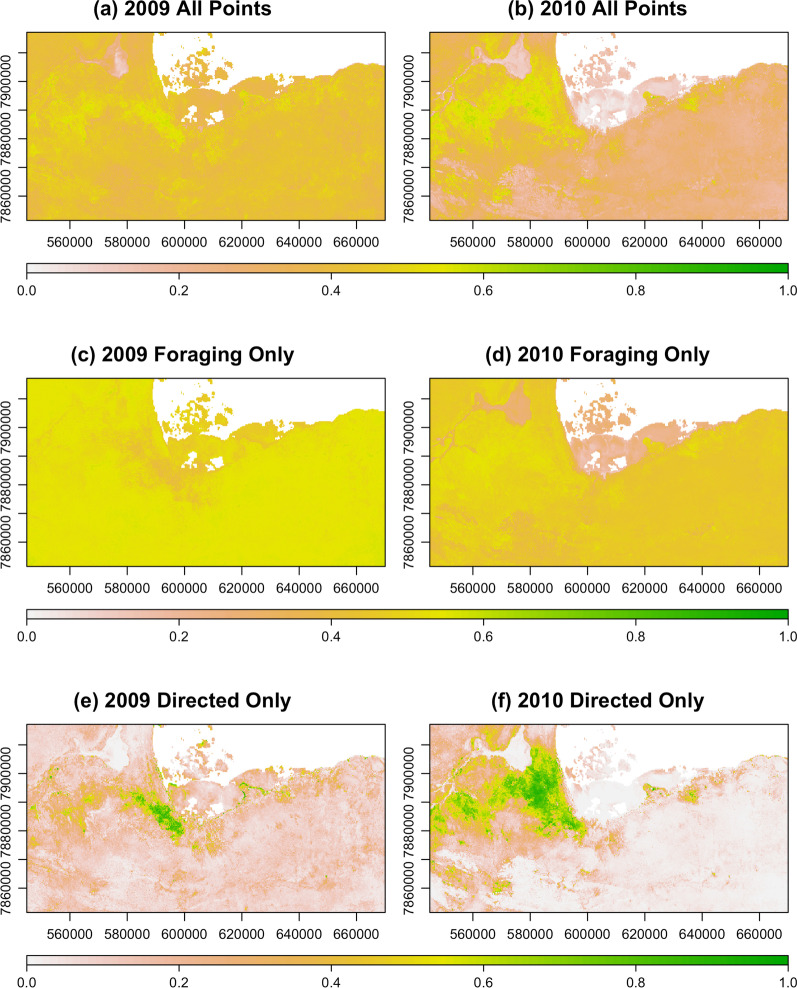
**Derived step-selection surface** Step selection functions within the region of interest in Etosha National Park, Namibia. Panels **a** and **b** illustrate the selection functions for anthrax seasons 2009 and 2010, respectively, when all of the recorded movement points are used. Panels **c** and **d** represent the selection functions during the same time periods, but using only the points during which the individual was in the foraging behavioral state. Panels **e** and **f** illustrate the selection surfaces when the animals were in the directed movement state in 2009 and 2010

For the sake of comparison, we first present the results of the analysis conducted using all of the movement points, irrespective of the behavioral state, followed by the results emerging from the analysis that explicitly incorporates behavior. In the latter case, we distinguished between the foraging and the directed movement state to determine how selection patterns compare across behavioral modes as well as across years (Fig. 3; Additional file 1: Table S18). Figure 4 provides a visual representation of the step selection function. This is a naïve mapping approach that should be interpreted somewhat differently from broader scale predictive outputs such as species distribution models: SSF heatmaps reflect likely past space use rather than predicted future use.

Comparing the 2009 and 2010 anthrax season SSFs suggest the following consistent trends. Wetness was the predictor with the largest effect on selection and was highly significant in terms of wetness avoidance both years (2009, $\beta _\mathrm{W} =$-0.52; $p < 0.001$; 2010, $\beta _\mathrm{W} =$-0.85; $p < 0.001$). In both years, individuals appeared to be slightly, though significantly, attracted to areas with greater Road Density (2009, $\beta _\mathrm{RD} = 0.01$, $p = 0.04$; 2010, $\beta _\mathrm{RD} = 0.03$, $p < 0.001$). Avoidance of areas with relatively high anthrax risk was consistent across years, and was close to significant in 2009 ($\beta _\mathrm{AR} = -0.02$, $p = 0.06$) and highly significant ($\beta _\mathrm{AR} = -0.06$; $p < 0.001$) in 2010. The only pattern that was not maintained across seasons was the role of Greenness. In 2009, Greenness was not a significant factor in patch selection ($p = 0.86$), though it was highly significant and second only in magnitude to Wetness in 2010 ($\beta _\mathrm{G} = 0.36$, $p < 0.001$).

When the dataset is parsed into different behavioral states, the results offer a slightly different picture, thereby providing some insight into the factors that animals consider when foraging rather than moving in a more directed manner. When considering only the foraging state, Wetness was no longer as consistent a predictor of habitat selection; being a highly significant negative factor in 2010 ($\beta _\mathrm{W} = -0.30$; $p < 0.001$), and a significant positive factor in 2009 ($\beta _\mathrm{W} = 0.23$; $p < 0.001$). In contrast, Greenness was the factor with the greatest impact on foraging phase movement decisions in 2010 ($\beta _\mathrm{G} = 0.43$; $p < 0.001$), but was not significant in 2009 ($p = 0.22$). Unlike the all-points analyses, the role of Road Density was negligible in both 2009 ($p = 0.61$) and 2010 ($p = 0.75$) during foraging. Importantly, in both years, zebra appear to consistently avoid the areas of highest risk of exposure to anthrax. Though the effect is slightly stronger in 2009 ($\beta _\mathrm{AR} = -0.11$, $p < 0.001$) than in 2010 ($\beta _\mathrm{AR} = -0.06$, $p < 0.001$), anthrax area avoidance behavior is highly significant across both seasons when animals are foraging.

Interrogating the selection patterns that emerge from an analysis of the directed movement points provides additional clarity compared with considering the foraging points alone. When animals move in a directed manner, with longer steps lengths and relatively little variance in their heading, they seem to actively avoid areas with high Wetness. The effect was highly significant in both 2009 ($\beta _\mathrm{W} = -2.17$, $p < 0.001$) and 2010 ($\beta _\mathrm{W} = -2.19$, $p < 0.001$). It was this large effect that likely drove the relatively high avoidance patterns when all of the points were analysed. There were several factors that exhibited notably different effects during the directed movement state than during the foraging state. In 2009, the effect of Greenness was negligible during the foraging state, but animals appeared to actively avoid areas of high Greenness during the directed movement state ($\beta _\mathrm{G} = -0.54$, $p < 0.001$). The oppositional trend was repeated in 2010, where foraging animals demonstrated a significant preference for higher Greenness, but animals moving in a directed manner seemed ambivalent to the level of Greenness ($p = 0.80$). Similarly, Road Density was a significant predictor of selection during directed movement in both 2009 ($\beta _\mathrm{RD} = 0.04$, $p = 0.001$) and 2010 ($\beta _\mathrm{W} = 0.07$, $p < 0.001$), where animals actively selected to be in areas with higher Road Density, but the factor did not appear to significantly affect movement decisions during the foraging state. A possible explanation for this difference is that roads may facilitate directed movement by eliminating potential barriers, making them more attractive for longer distance ‘steps’. However, the disparity between the behavioral states was perhaps most notable with regard to role of disease risk in movement decisions. When animals were moving in a directed manner, they consistently selected areas that correlated with greater risk of exposure, whereas animals tended to actively avoid such high risk areas when they were foraging. This was the case in 2009 ( $\beta _\mathrm{AR}^\mathrm{direct}= 0.13$, $p = 0.003$ versus $\beta _\mathrm{AR}^\mathrm{forage}= -0.11$, $p < 0.001$) and in 2010 ($\beta _\mathrm{AR}^\mathrm{direct}= 0.09$, $p < 0.001$ versus $\beta _\mathrm{AR}^\mathrm{forage}= -0.06$, $p < 0.001$).

## Discussion

The selection functions revealed an interesting, and somewhat unexpected, dynamic with regard to the behaviorally-contingent space use patterns of the zebra. An analysis that neglected the internal state of the animal by analyzing the full track would likely overlook the contrasting selection patterns that emerged in both 2009 and 2010 when the behavioral state was used to guide the analysis. In both years examined here, the zebra exhibit apparent avoidance (indicated by a negative selection coefficient) of high-risk areas as defined in our risk map of *Bacillus anthracis* persistence (Fig. 1) when they are in the foraging state, and a pattern of attraction (indicated by a positive selection coefficient) to these areas of high risk when they are in the directed movement state. Importantly, this does not necessarily suggest that the animals are aware of anthrax risk nor that they are actively choosing a strategy that reduces their likelihood of exposure. In fact, the observed trend may very well be an artifact of other features of the landscape that were not captured by the predictor variables used in the SSF.

It is also important to note that these general trends do not indicate that animals never forage in areas with some risk of exposure; it merely implies that animals exhibit a statistically meaningful preference for areas with lower over higher risk while in the foraging state. Similarly, zebra will, on occasion, move in a directed manner outside of high risk areas, but there exists a meaningful preference such that they are more likely to select for areas of high versus low risk when they are in the directed movement state.

The association with directed movements occurring in the high-risk area may suggest that exposure in this area is less than it could be if foraging behavior occurred here more often. The reduction in foraging behavior may be due to overgrazing depleting the desirable short-grass resource in this area, forcing animals to forage elsewhere [29, 35, 66]. An alternative interpretation of this association with directed movements could be evidence of increased anthrax mortality occurring during the directed movement state. There is a time lag between host exposure and anthrax mortality, estimated to be several days [67]. Zebras in this system move 13 to 16 kilometers per day on average (depending on the season and whether they are members of a migratory or non-migratory herd; [30]), blurring the lines between where higher risk behaviors occur and where disease mortalities are detected. Although little is known about post-exposure behaviors in wildlife, anthrax infected hippos in Tanzania showed no changes in their movement patterns in the days leading up to mortality [68].

It is beyond the scope of this methodologically-focused discussion, however, to establish the mechanisms underlying the patterns observed here. Rather, our intention is to draw attention to the generally contradictory selection patterns that emerge during two distinct behavioral states, which are largely obscured when behavioral state is ignored. Even so, we emphasize below a number of caveats that are important to consider when conducting and interpreting analyses following the general structure outlined here. We also note that, though the focus of our discussion has been on the avoidance of pathogens that are transmitted from an environmental reservoir, some of the ideas we present can be applied to other types of transmission [21], such as vector systems (e.g., tick or mosquito borne diseases) or direct transmission between live individuals (e.g., bovine tuberculosis). In such cases it may actually be easier to detect avoidance behavior in movement trajectories because the entities being avoided may be directly observable (e.g., tracking the population densities of vectors or infected heterospecifics).

The value of this analytical framework lies in the ability—and need—to parse continuous movement tracks into relevant and separable behavioral states. The selection of a particular analytical method to classify portions of the movement track into the canonical activity modes (CAMs; [3, 69]) or behavioral states can introduce uncertainty from the outset. For this study, we have chosen the HMM approach that accounts for some of this uncertainty by probabilistically assigning each point to a given state. However, the models that emerge from this method are highly dependent on user inputs, including an *a priori* decision regarding the number of states to which points can be assigned. This is an important consideration when researchers intend to incorporate behavior into models of habitat selection.

Recently, telemetry devices have been fitted with auxiliary sensors, such as accelerometers [4, 70], that might offer additional clarity to researchers wishing to parse movement tracks. Similarly, several new tracking devices directly account for the movement mode of an animal by altering the positional fix rate based on the current speed of movement, offering classification of steps without additional analyses [20]. However, it is unclear exactly what the ramifications of misclassification would be, and the identification of these effects will be difficult without definitive knowledge of the actual behavioral states of an animal through time.

The pathogen persistence map, upon which our interpretation of habitat usage patterns is based, suffered from data limitations. An essential component to modeling disease risk for an environmentally transmitted disease is a good understanding of the heterogeneity in pathogen deposition into the environment from infected hosts, and variation in pathogen survival in different habitats or environmental conditions. In theory, with enough reliable data, the method proposed here would enable a direct mapping of suitability for spore persistence, and thus, exposure risk, as opposed to mapping some proxy, such as habitat preferences of animals weakened by anthrax infection.

Despite building our analysis on one of the most extensive datasets on *B. anthracis* persistence available, this dataset is of a relatively small spatial extent compared to the areas used by hosts, with few of the monitored sites showing low or no survival of the pathogen, which may affect the inferences made in this study. Results from a reciprocal transplant experiment of *B. anthracis* spore persistence in ENP soils suggest that soils from across the park are suitable for spore persistence [71], thus our pseudo-absences may not accurately reflect an absence of the pathogen. Although no small lift to acquire, a detailed pathogen risk layer is important for evaluating disease transmission risk, especially one that combines both a large spatial extent and sufficient detail at the small scale where hosts encounter individual pathogen reservoirs. Because the risk map itself was not the primary focus of this effort, we decided to expand the range over which we placed our pseudo-absence points to more closely match the range of our movement data rather than the pathogen data. Ideally, a more representative area would be sampled by the pseudo-absence points or a much broader area would be surveyed to build the presence dataset.

Another potential source of uncertainty in the interpretation of such an analysis is the specific scales at which the data were collected and subsequently analysed. The locally infectious zone (LIZ; [37]) generated by a zebra carcass is, on average, only 2-3 meters in diameter. Thus, it is not especially surprising that an analysis at a 30 meter resolution could miss some of the finer-scale dynamics. At this scale, the signature of a LIZ site is likely overwhelmed by the averaging of the characteristics of a cell (the LIZ likely represents only about one-tenth of the area of a cell at this scale, though this depends on the species of the animal that succumbed at that location). Thus, at the scale of this analysis, animals may, in fact, select for areas that present lower risk of exposure to a pathogen.

Avoidance behavior has been noted in other species [72, 73], and has given rise to the concept of the ‘landscape of disgust’ [74]. Although whether or not hosts can detect and avoid bacterial pathogens such as *B. anthracis* in the environment, beyond relying on indirect cues such as the carcass, is unknown. Evidence from an anthrax-endemic system in Montana, USA, where bison and elk graze near carcasses soon after death suggests that not all species demonstrate behaviors indicative of ‘disgust’ [75]. If an animal ends up in a high risk cell, they might be attracted to the LIZ site within that cell due to vegetation green-up at the carcass site [32]. Thus, an overall avoidance pattern may be observed across the landscape, with attraction at the sub-cell scale. This possibility implies a very important point about habitat selection analyses that are frequently conducted at the finest scale allowed by the environmental data, as opposed to the most meaningful scale from a biological perspective. It should also be noted, however, that to make use of such fine-scale environmental data, the temporal resolution of the movement tracks would also likely need to be finer, perhaps on the order of 1 minute per fix. At the time that these data were collected in 2009 and 2010, this technology was not widely available, but recent advancements in GPS devices make such fine-scale movement data more readily collectable. Even so, a trade-off still exists between tracking period and fix rate, so careful consideration of the primary goals of the study are required [76].

## Conclusion

In conclusion, we applied a somewhat unconventional approach to our habitat selection modeling. The methods outlined by [26] offer an alternative that overcomes several of the shortcomings of conventional resource- and step-selection functions, and arguably reflects the selection process in a more biologically accurate fashion. At the same time, the approach foregoes some of the established statistical characteristics of conventional SSFs [25] by evaluating a summary of all of the resources theoretically reachable by the individual. The effects of the SSF approach were not explicitly compared to more conventional approaches here but warrant further investigation.

The explicit consideration of particular behavioral states in habitat selection studies can offer important insights, especially in systems with environmentally-transmitted pathogens. The unique biology of these pathogens enables them to persist in reservoirs outside of hosts for relatively long periods of time. Anthrax spores, for example, may remain viable in the soil in Etosha for at least seven years [33]; it is possible that they can persist even longer in systems with more vegetation cover, potentially giving rise to episodic infection dynamics [22]. Where environmental persistence is possible, our ability to predict the presence of the pathogen is directly related to the dependence of the pathogen on particular environmental factors. Broadly, *B. anthracis* exhibits a dependence on soil with a slightly alkaline pH, relatively high organic matter, and high calcium content [77]. The availability of these remotely sensed data makes it feasible to predict the potential distribution of the pathogen, and anthrax risk, within a niche modeling framework.

Though other disease systems, including those characterized by transmission via environmental reservoirs, might not involve a particular behavioral state that exhibits a definitively higher level of vulnerability, the consideration of behavior could be important for judging other epidemiological processes, such as contact or succumbing to infection [6]. In the case of the former, particular behavioral modes might result in shifting selection patterns that lead to large aggregations of individuals, thereby placing animals at a higher risk of contacting an infectious conspecific [78]. When investigating the infection process itself, novel selection patterns may be induced by infection with a parasite or pathogen, and these shifts might be apparent in a movement track [79, 80]. The growing availability of fine-scale GPS data and the growing set of analytical methods to infer behavior from such data makes the direct incorporation of behavior an important and exciting avenue for future exploration.

## Supplementary Information

**Additional file 1**. Contains additional details on several of the methods undertaken here. These intricacies are outside of the scope of the article presented here, but may be useful in replicating the approach.

**Additional file 2**. Contains supplementary tables, including individual zebra HMM outputs, details extracted from the MaxEnt modeling process and input feature selection, and results from the conditional logistic regression model.

**Additional file 3**. Contains supplementary figures illustrating the soil, bioclimatic, and vegetation covariate layers used in the anthrax suitability modeling.

## Data Availability

Please contact Eric R Dougherty (dougherty.eric@berkeley.edu) for data requests.

## Declarations

### Trade disclaimer

Any use of trade, firm, or product names is for descriptive purposes only and does not imply endorsement by the U.S. Government.
