# Correction: A framework for integrating inferred movement behavior into disease risk models

**DOI:** 10.1186/s40462-022-00334-5

**Published:** 2022-08-25

**Authors:** Eric R. Dougherty, Dana P. Seidel, Jason K. Blackburn, Wendy C. Turner, Wayne M. Getz

**Affiliations:** 1 Department of Environmental Science, Policy, and Management, University of California Berkeley, Berkeley, CA USA; 2 Spatial Epidemiology and Ecology Research Laboratory, Department of Geography, University of Florida, Gainesville, FL USA; 3 Emerging Pathogens Institute, University of Florida, Gainesville, FL USA; 4 U.S. Geological Survey, Wisconsin Cooperative Wildlife Research Unit, Department of Forest and Wildlife Ecology, University of Wisconsin-Madison, Madison, WI USA; 5 School of Mathematical Sciences, University of KwaZulu-Natal, Durban, South Africa

## Correction to: Movement Ecology (2022) 10:31. https://doi.org/10.1186/s40462-022-00331-8

Following publication of the original article [1], it was noted that the Additional file citations in the body of the article were incorrect. The citations to the supplementary tables should refer to Additional file 2 instead of Additional file 1 and the citations to the supplementary figures should refer to Additional file 3 instead of Additional file 1. In addition, the citation to the Supplementary Materials should be changed to a citation to Additional file 1.

The original article [1] has been corrected.
