# Supplementary material for: A framework for integrating inferred movement behavior into disease risk models
Source: Mov Ecol. 2022 Jul 24;10:31. doi: 10.1186/s40462-022-00331-8 (PMC9310477; doi:10.1186/s40462-022-00331-8)
Supplement: Supplementary file 1 — Additional file 1. Contains additional details on several of the methods undertaken here. These intricacies are outside of the scope of the article presented here, but may be useful in replicating the approach. [file 40462_2022_331_MOESM1_ESM.pdf]

Supplementary Materials

Environmental predictor derivation

Several potential predictor layers were used in constructing the step-selection functions. Three continuous variables were derived directly from the available bands of the Landsat 4-5 Thematic Mapper remote sensing data. The Normalized Difference Vegetation Index (NDVI) was calculated using the standard formula:

$$\text{NDVI} = \frac{\text{NIR} - \text{Red}}{\text{NIR} + \text{Red}}$$

where Red is Band 4 (B_4) and NIR is Band 5 (B_5). Greenness (G) and Wetness (W) were calculated based on the tasseled-cap transformation equation presented by [1], which utilizes 6 of the 7 bands in a regression framework to calculate several measures (weights rounded to 2 decimal places):

$$G = -0.29B_1 - 0.24B_2 - 0.54B_3 + 0.72B_4 + 0.08B_5 - 0.18B_7$$

$$W = 0.15B_1 + 0.18B_2 + 0.33B_3 + 0.34B_4 - 0.71B_5 - 0.46B_7$$

These measures were calculated for the four cloudless images available during the 2009 anthrax season (March 22, April 23, May 9, and May 25) and the four cloudless images available during the 2010 anthrax season (February 5, April 10, May 12, and May 28). A single mean layer was then calculated for the Greenness and Wetness metrics in each year. All of these layers were obtained at a resolution of 30 meters.

The mean Wetness and mean NDVI measures were highly (negatively) correlated at the landscape scale in the region through which the zebra moved ($R = -0.95$ for the 2009 season and $R = -0.94$ for the 2010 season), and the latter was eliminated from subsequent analyses. The correlations between these mean Greenness and mean Wetness at the landscape scale were relatively low ($R = -0.59$ in 2009 and $R = -0.47$ in 2010), so both were maintained as potential predictors.

To account for the potential impact of human development in the area, primary roads (i.e., those made of tar or gravel) were mapped and recast in the form of a road density layer. The road density layer was created in ArcMap 10.3.1 by calculating the length of road (in meters) per unit area (square meters) in each 30 meter raster cell. This layer exhibited low covariance with the other two that were maintained in the predictor variable set, resulting in a total of three continuous predictor variables. Other frequently used continuous variables, such as elevation, slope, and aspect, were eliminated *a priori* due to the natural homogeneity of the study site with regard to those variables. Though potentially important, particularly in terms of aggregating anthrax spores [2], the minute differences in elevation across Etosha National Park were not detectable at the resolution at which data were available.

Selection of environmental prediction layers

For all three categories (soil characteristics, bioclimatic variables, and vegetation indices), sets of variables were initially selected based on associations with *B. anthracis* survival in other modeling studies. The soil component was summarized using soil pH in H_2O (pH), organic carbon content (OC), and cation exchange capacity (CEC). All three of these layers were obtained from the SoilGrids database at a resolution of 250m. The five bioclimatic variables consisted of mean annual temperature (bio1), mean temperature range (bio7), annual precipitation (bio12), precipitation of the wettest month (bio13) and precipitation of the driest month (bio14). The latter variable was removed because it was uninformative in the region of interest (i.e., every cell had the same value). The bioclimatic variable layers were obtained from the WorldClim database

at a resolution of 30 arc-seconds ($\approx$1 km). The potential vegetation indices were calculated based on the Landsat 7 NDVI 8-Day Composite layers, which were obtained at a resolution of 30m. Due to the disparity in resolution, the soil characteristic and bioclimatic variables were upsampled using bilinear interpolation. This enabled compatibility between the low resolution bioclimatic data and the higher resolution vegetation data. Because spore persistence may exhibit dependence on local vegetation patterns during the entire period of sampling (i.e., the year of deposition and the two subsequent years), the vegetation metrics were calculated for the three year periods between initial discovery and final sampling. The vegetation indices that served as potential predictors were the mean NDVI (mean_ndvi), maximum NDVI (max_ndvi), minimum NDVI (min_ndvi), and the range of NDVI over the period of observation (range_ndvi). To minimize the covariance between predictor variables used in the model for each year, a Pearson correlation matrix was calculated, and any pairs of variables whose coefficient of correlation was > 0.8 were reduced to a single variable as explained below.

MaxEnt modeling details

To parameterize a MaxEnt model, the background distribution of predictors must be considered. This requires a sampling protocol in which points are randomly dispersed throughout the region of interest and the values of the environmental predictors at those points (i.e., pseudo-absence points) are recorded for comparison with the presence points. In this case, 500 background points were selected. Due to the association of carcasses with a particular deposition year, these background points were divided into three groups in proportion to the number of observed carcasses in each of the three years (2010, 2011, 2012). The result was a set of pseudo-absence points consisting of 312, 50, and 138 points associated with 2010, 2011, and 2012 seasons, respectively. The predictor values were extracted for each presence and pseudo-absence point according to its deposition year. An initial MaxEnt model was run on these data and the full candidate predictor set using the implementation in the `dismo` package (version 1.1-4; [3]) in R (version 3.4.3; [4]). Following an investigation of the variable contributions to this full model (generated as a standard output of the `maxent` function), variables exhibiting covariance with another predictor were culled such that the variable in the pair with the higher contribution to the MaxEnt model was maintained and its counterpart eliminated. Finally, another MaxEnt model was run on the reduced predictor variable set.

Carcass dataset robustness

To verify that the reduced carcass dataset was sufficient for the derivation of the anthrax risk layer, two alternative approaches were applied. The first was a simple calculation of the area under the receiver operating characteristic (ROC) curve using the comprehensive set of observed anthrax carcasses from 2009 and 2010. The total number of test points in this set was 237 (69 from 2009 and 168 from 2010). The second approach involved the use of Warren's I metric [5]. This metric, which ranges in value from 0 (indicating no overlap) to 1 (complete overlap), aims to measure niche equivalency using the output raster layers of species distribution models. Here, 100 separate niche models were created using random selections of 40 points from the full carcass dataset (including both the set of 40 sites where anthrax concentrations were measured in subsequent years and the set of 237 observed carcasses from 2009 an 2010). In addition, 500 pseudo-absence points were randomly assigned from a set of 1149. The resulting MaxEnt model based on each random subset of the presence and pseudo-absence data was projected onto the predictor layers from the 2009 and 2010 seasons, and the suitability maps were compared to the final maps derived based on only the 40 presence points at which the concentration of anthrax was measured in subsequent years. The Warren's I metric was then calculated for both the 2009 and 2010 season to determine the similarity and judge the efficacy of using the reduced dataset on *B. anthrasis* persistence rather than carcass presence.

The final model had an AUC value of 0.94 on the training data. When the AUC was calculated using the full dataset of 237 observed carcasses from 2009 and 2010 as a test set, the AUC was 0.92. It is not unexpected for the AUC of the training set to be higher than that of the testing set, so this was deemed an acceptable AUC value. In addition, the mean Warren's I metric between the final anthrax suitability map during the 2009 season and the 100 alternative suitability maps built based on the full carcass dataset was 0.88. In 2010, the mean Warren's I metric across the 100 alternative maps was 0.90. Both of these values indicate a relatively high correspondence between alternative subsets of the data and support the use of the reduced dataset ultimately used to construct the MaxEnt model here.

Author details

References

1. Crist, E.P., Cicone, R.C.: A physically-based transformation of thematic mapper data—the tm tasseled cap. IEEE Transactions on Geoscience and Remote sensing (3), 256–263 (1984)
2. Dragon, D., Bader, D., Mitchell, J., Woollen, N.: Natural dissemination of bacillus anthracis spores in northern canada. Applied and environmental microbiology **71**(3), 1610–1615 (2005)
3. Hijmans, R.J., Phillips, S., Leathwick, J., Elith, J.: Dismo: Species Distribution Modeling. (2017). R package version 1.1-4. https://CRAN.R-project.org/package=dismo
4. R Core Team: R: A Language and Environment for Statistical Computing. R Foundation for Statistical Computing, Vienna, Austria (2017). R Foundation for Statistical Computing. https://www.R-project.org/
5. Warren, D.L., Glor, R.E., Turelli, M.: Environmental niche equivalency versus conservatism: quantitative approaches to niche evolution. Evolution **62**(11), 2868–2883 (2008)
