# Supplementary material for: A framework for integrating inferred movement behavior into disease risk models
Source: Mov Ecol. 2022 Jul 24;10:31. doi: 10.1186/s40462-022-00331-8 (PMC9310477; doi:10.1186/s40462-022-00331-8)
Supplement: Supplementary file 2 — Additional file 2. Contains supplementary tables, including individual zebra HMM outputs, details extracted from the MaxEnt modeling process and input feature selection, and results from the conditional logistic regression model. [file 40462_2022_331_MOESM2_ESM.pdf]

Supplementary Tables

Table 1 HMM results for AG059 during 2009 season

Behavioral State	Step Lengths (Gamma)*		Turning Angles (vonMises)	
	μ (m)	σ (m)	mean (radians)	concentration
Resting	23.5	27.6	-2.5	0.03
Foraging	193.6	178.6	0.01	1.8
Directed	853.8	299.0	-0.01	20.5

*Gamma distributions may be parameterized with an additional zero-mass value, but these are excluded here because their magnitude was negligible in all models

Table 2 HMM results for AG061 during 2009 season

Behavioral State	Step Lengths (Gamma)*		Turning Angles (vonMises)	
	μ (m)	σ (m)	mean (radians)	concentration
Resting	6.0	4.7	-3.0	0.27
Foraging	78.3	73.4	0.02	0.89
Directed	405.8	306.8	0.01	3.8

*Gamma distributions may be parameterized with an additional zero-mass value, but these are excluded here because their magnitude was negligible in all models

Table 3 HMM results for AG062 during 2009 season

Behavioral State	Step Lengths (Gamma)*		Turning Angles (vonMises)	
	μ (m)	σ (m)	mean (radians)	concentration
Resting	2.9	2.1	2.9	0.28
Foraging	68.0	64.7	-0.02	0.57
Directed	386.4	297.0	0.01	2.6

*Gamma distributions may be parameterized with an additional zero-mass value, but these are excluded here because their magnitude was negligible in all models

Table 4 HMM results for AG063 during 2009 season

Behavioral State	Step Lengths (Gamma)*		Turning Angles (vonMises)	
	μ (m)	σ (m)	mean (radians)	concentration
Resting	30.7	36.4	-0.15	0.17
Foraging	188.1	151.8	0.01	1.8
Directed	758.0	332.0	-0.03	5.6

*Gamma distributions may be parameterized with an additional zero-mass value, but these are excluded here because their magnitude was negligible in all models

Table 5 HMM results for AG068 during 2009 season

Behavioral State	Step Lengths (Gamma)*		Turning Angles (vonMises)	
	μ (m)	σ (m)	mean (radians)	concentration
Resting	11.5	13.1	-2.97	0.15
Foraging	173.4	163.0	0.03	1.4
Directed	907.7	304.5	-0.01	12.3

*Gamma distributions may be parameterized with an additional zero-mass value, but these are excluded here because their magnitude was negligible in all models

Table 6 HMM results for AG063 during 2010 season

Behavioral State	Step Lengths (Gamma)*		Turning Angles (vonMises)	
	μ (m)	σ (m)	mean (radians)	concentration
Resting	23.9	27.4	-3.01	0.1
Foraging	205.6	163.0	< 0.01	1.4
Directed	818.9	369.5	0.01	4.1

*Gamma distributions may be parameterized with an additional zero-mass value, but these are excluded here because their magnitude was negligible in all models

Table 7 HMM results for AG068 during 2010 season

Behavioral State	Step Lengths (Gamma)*		Turning Angles (vonMises)	
	μ (m)	σ (m)	mean (radians)	concentration
Resting	24.2	30.2	-1.26	0.02
Foraging	187.0	155.0	0.04	1.4
Directed	852.0	339.9	-0.02	4.1

*Gamma distributions may be parameterized with an additional zero-mass value, but these are excluded here because their magnitude was negligible in all models

Table 8 HMM results for AG252 during 2010 season

Behavioral State	Step Lengths (Gamma)*		Turning Angles (vonMises)	
	μ (m)	σ (m)	mean (radians)	concentration
Resting	17.6	16.5	0.17	0.27
Foraging	110.9	88.9	-0.01	1.6
Directed	600.1	348.1	< 0.01	3.1

*Gamma distributions may be parameterized with an additional zero-mass value, but these are excluded here because their magnitude was negligible in all models

Table 9 HMM results for AG253 during 2010 season

Behavioral State	Step Lengths (Gamma)*		Turning Angles (vonMises)	
	μ (m)	σ (m)	mean (radians)	concentration
Resting	24.5	24.6	0.17	0.27
Foraging	154.1	131.8	-0.01	1.6
Directed	785.5	385.5	0.01	2.4

*Gamma distributions may be parameterized with an additional zero-mass value, but these are excluded here because their magnitude was negligible in all models

Table 10 HMM results for AG255 during 2010 season

Behavioral State	Step Lengths (Gamma)*		Turning Angles (vonMises)	
	μ (m)	σ (m)	mean (radians)	concentration
Resting	5.49	4.65	3.07	0.41
Foraging	97.1	86.1	-0.04	0.89
Directed	560.1	376.3	-0.01	3.7

*Gamma distributions may be parameterized with an additional zero-mass value, but these are excluded here because their magnitude was negligible in all models

Table 11 HMM results for AG256 during 2010 season

Behavioral State	Step Lengths (Gamma)*		Turning Angles (vonMises)	
	μ (m)	σ (m)	mean (radians)	concentration
Resting	5.98	5.69	3.09	0.30
Foraging	108.4	100.9	0.03	1.1
Directed	581.8	362.9	0.01	3.5

*Gamma distributions may be parameterized with an additional zero-mass value, but these are excluded here because their magnitude was negligible in all models

Table 12 Pearson Correlation matrix among predictor variables for carcasses deposited in 2010

	**pH**	**OCC**	**CEC**	**bio1**	**bio7**	**bio12**	**bio13**	**mean**	**max**	**min**	**range**
**pH**	1	0.32	0.46	-0.12	0.23	-0.50	-0.52	-0.40	-0.22	-0.17	-0.15
**OCC**	0.32	1	0.45	0.04	0.25	-0.18	-0.19	-0.35	-0.33	-0.22	-0.26
**CEC**	0.46	0.45	1	0.21	0.43	-0.30	-0.30	-0.39	-0.24	-0.21	-0.15
**bio1**	-0.12	0.04	0.21	1	0.50	-0.17	-0.11	-0.07	0.00	-0.02	0.01
**bio7**	0.23	0.25	0.43	0.50	1	-0.24	-0.26	-0.46	-0.31	-0.24	-0.20
**bio12**	-0.50	-0.18	-0.30	-0.17	-0.24	1	0.98	0.02	-0.14	-0.14	-0.07
**bio13**	-0.52	-0.19	-0.30	-0.11	-0.26	0.98	1	0.03	-0.16	-0.13	-0.10
**mean_ndvi**	-0.40	-0.35	-0.39	-0.07	-0.46	0.02	0.03	1	0.89	0.80	0.49
**max_ndvi**	-0.22	-0.33	-0.24	0.00	-0.31	-0.14	-0.16	0.89	1	0.71	0.74
**min_ndvi**	-0.17	-0.22	-0.21	-0.02	-0.24	-0.14	-0.13	0.80	0.71	1	0.05
**range_ndvi**	-0.15	-0.26	-0.15	0.01	-0.20	-0.07	-0.10	0.49	0.74	0.05	1

Table 13 Pearson Correlation matrix among predictor variables for carcasses deposited in 2011

	pH	OCC	CEC	bio1	bio7	bio12	bio13	mean	max	min	range
**pH**	1	0.32	0.46	-0.12	0.23	-0.50	-0.52	-0.41	-0.21	-0.16	-0.15
**OCC**	0.32	1	0.45	0.04	0.25	-0.18	-0.19	-0.34	-0.30	-0.22	-0.22
**CEC**	0.46	0.45	1	0.21	0.43	-0.30	-0.30	-0.41	-0.23	-0.16	-0.18
**bio1**	-0.12	0.04	0.21	1	0.50	-0.17	-0.11	-0.10	-0.04	0.03	-0.07
**bio7**	0.23	0.25	0.43	0.50	1	-0.24	-0.26	-0.50	-0.33	-0.21	-0.26
**bio12**	-0.50	-0.18	-0.30	-0.17	-0.24	1	0.98	0.06	-0.15	-0.08	-0.13
**bio13**	-0.52	-0.19	-0.30	-0.11	-0.26	0.98	1	0.07	-0.17	-0.06	-0.17
**mean_ndvi**	-0.41	-0.34	-0.41	-0.10	-0.50	0.06	0.07	1	0.85	0.72	0.57
**max_ndvi**	-0.21	-0.30	-0.23	-0.04	-0.33	-0.15	-0.17	0.85	1	0.61	0.83
**min_ndvi**	-0.16	-0.22	-0.16	0.03	-0.21	-0.08	-0.06	0.72	0.61	1	0.06
**range_ndvi**	-0.15	-0.22	-0.18	-0.07	-0.26	-0.13	-0.17	0.57	0.83	0.06	1

Table 14 Pearson Correlation matrix among predictor variables for carcasses deposited in 2012

	pH	OCC	CEC	bio1	bio7	bio12	bio13	mean	max	min	range
**pH**	1	0.32	0.46	-0.12	0.23	-0.50	-0.52	-0.44	-0.30	-0.20	-0.26
**OCC**	0.32	1	0.45	0.04	0.25	-0.18	-0.19	-0.34	-0.29	-0.26	-0.22
**CEC**	0.46	0.45	1	0.21	0.43	-0.30	-0.30	-0.42	-0.24	-0.21	-0.18
**bio1**	-0.12	0.04	0.21	1	0.50	-0.17	-0.11	-0.09	0.02	0.07	-0.01
**bio7**	0.23	0.25	0.43	0.50	1	-0.24	-0.26	-0.50	-0.31	-0.21	-0.26
**bio12**	-0.50	-0.18	-0.30	-0.17	-0.24	1	0.98	0.06	-0.13	-0.04	-0.13
**bio13**	-0.52	-0.19	-0.30	-0.11	-0.26	0.98	1	0.07	-0.13	0.00	-0.15
**mean_ndvi**	-0.44	-0.34	-0.42	-0.09	-0.50	0.06	0.07	1	0.87	0.66	0.71
**max_ndvi**	-0.30	-0.29	-0.24	0.02	-0.31	-0.13	-0.13	0.87	1	0.59	0.90
**min_ndvi**	-0.20	-0.26	-0.21	0.07	-0.21	-0.04	0.00	0.66	0.59	1	0.18
**range_ndvi**	-0.26	-0.22	-0.18	-0.01	-0.26	-0.13	-0.15	0.71	0.90	0.18	1

Table 15 Variable contribution and importance results from the full MaxEnt model, built on the full environmental predictor set. Due to covariance observed in Tables S13-S15, two pairs of predictors were considered for variable set reduction: bio12 with bio13 and mean_ndvi with max_nndvi.

Variable	Name	Percent contribution	Permutation importance
Mean temperature range	bio7	72.6	71.5
Soil Organic Carbon Content	OC	11.3	2.6
Precipitation of the wettest month	bio13	5.3	15.5
Range of NDVI	range_ndvi	4.7	0.9
Maximum NDVI	max_ndvi	2.4	1.5
Annual precipitation	bio12	2	4.2
Mean annual temperature	bio1	1.1	0.8
Soil pH	pH	0.5	0.7
Soil Cation Exchange Efficiency	CEC	0.1	1.7
Minimum NDVI	min_ndvi	0	0.8
Mean NDVI	men_ndvi	0	0

Table 16 Final MaxEnt model ‘lambda’ values associated with the variables (and their derivatives) ultimately included.

Variable	Lambda	Min	Max
CEC	0.222	7.075	26.000
OC	-5.052	0.000	58.429
bio1	0.669	22.225	23.194
bio13	3.481	86.000	116.000
bio7	8.804	27.285	30.536
max_ndvi	0.000	0.092	0.639
min_ndvi	3.044	-0.309	0.160
pH	0.000	72.655	83.327
range_ndvi	0.000	0.087	0.589
CEC^2	2.560	50.052	676.000
bio1^2	0.522	493.929	537.960
bio7^2	2.072	744.498	932.440
‘OC	-1.015	0.000	0.350
‘bio13	-0.369	86.000	95.000
’range_ndvi	-1.278	0.382	0.589
’bio1	-0.421	23.093	23.194
’bio1	-0.546	23.092	23.194
’bio7	-1.240	30.280	30.536
’range_ndvi	-0.324	0.382	0.589
‘pH	-0.882	72.655	78.347
‘max_ndvi	-1.746	0.092	0.355
’bio1	-0.183	23.090	23.194
‘OC	-0.180	0.000	1.008
’bio7	-0.525	30.209	30.536
’bio1	-0.271	23.060	23.194
’bio7	-0.173	30.205	30.536
‘bio13	-0.178	86.000	95.262
‘OC	-0.229	0.000	1.003

var^2 represents quadratic feature

’var represents forward hinge feature
‘var represents reverse hinge feature
linearPredictorNormalizer: 15.142
densityNormalizer: 34.768
entropy: 4.561

Table 17 Results of the conditional logistic mixed effects models as applied to all of the movement points in 2010 ($n = 56495$) including two randomly generated predictor layers (Rand and Rand2) to evaluate the efficacy of the step-selection methodology employed throughout.

**2010 All Points**	coef	exp(coef)	se(coef)	z	p	sig
Rand	-0.00	1.00	0.009	-0.37	0.71	
Rand2	-0.01	0.99	0.009	-1.15	0.25	
Wet_Norm	-0.85	0.43	0.023	-36.57	<2e-16	***
Green_Norm	0.36	1.43	0.031	11.40	<2e-16	***
Road_Dens_Norm	0.03	1.03	0.004	6.16	7.1e-10	***
Risk_Norm	-0.06	0.94	0.008	-7.56	3.9e-14	***

Table 18 Results of the conditional logistic mixed effects models as applied to all of the movement points (n = 22,949 in 2009 and n= 56,495 in 2010), only the foraging points (n = 11,733 in 2009 and n = 27,898 in 2010), and only the directed movement points (n = 4,381 in 2009 and n = 11,486 in 2010).

**2009 All Points**	coef	exp(coef)	se(coef)	z	p	sig
Wetness (β_{W})	-0.52	0.59	0.039	-13.26	<2e-16	***
Greenness (β_{G})	-0.01	0.99	0.031	-0.18	0.86	
Road Density (β_{RD})	0.01	1.01	0.006	2.03	0.04	*
Anthrax Risk (β_{AR})	-0.02	0.98	0.012	-1.91	0.06	.

**2010 All Points**	coef	exp(coef)	se(coef)	z	p	sig
Wetness (β_{W})	-0.85	0.43	0.023	-36.58	<2e-16	***
Greenness (β_{G})	0.36	1.43	0.031	11.38	<2e-16	***
Road Density (β_{RD})	0.03	1.03	0.004	6.16	7.1e-10	***
Anthrax Risk (β_{AR})	-0.06	0.95	0.008	-7.57	3.8e-14	***

**2009 Foraging Points**	coef	exp(coef)	se(coef)	z	p	sig
Wetness (β_{W})	0.23	1.25	0.052	4.34	1.4e-05	***
Greenness (β_{G})	0.05	1.05	0.042	1.22	0.22	
Road Density (β_{RD})	-0.00	1.00	0.008	-0.50	0.61	
Anthrax Risk (β_{AR})	-0.11	0.90	0.018	-6.05	1.4e-09	***

**2010 Foraging Points**	coef	exp(coef)	se(coef)	z	p	sig
Wetness (β_{W})	-0.30	0.74	0.030	-9.98	<2e-16	***
Greenness (β_{G})	0.43	1.54	0.044	9.88	<2e-16	***
Road Density (β_{RD})	-0.00	1.00	0.006	-0.31	0.75	
Anthrax Risk (β_{AR})	-0.06	0.94	0.011	-5.85	4.8e-09	***

**2009 Directed Points**	coef	exp(coef)	se(coef)	z	p	sig
Wetness (β_{W})	-2.17	0.11	0.145	-14.97	<2e-16	***
Greenness (β_{G})	-0.54	0.58	0.130	-4.15	3.3e-05	***
Road Density (β_{RD})	0.04	1.04	0.012	3.26	0.001	**
Anthrax Risk (β_{AR})	0.13	1.14	0.043	3.01	0.003	**

**2010 Directed Points**	coef	exp(coef)	se(coef)	z	p	sig
Wetness (β_{W})	-2.19	0.11	0.075	-29.37	<2e-16	***
Greenness (β_{G})	-0.02	0.98	0.097	-0.25	0.80	
Road Density (β_{RD})	0.07	1.07	0.008	8.36	<2e-16	***
Anthrax Risk (β_{AR})	0.09	1.09	0.024	3.81	1.4e-04	***
