# Supplementary material for: A framework for integrating inferred movement behavior into disease risk models
Source: Mov Ecol. 2022 Jul 24;10:31. doi: 10.1186/s40462-022-00331-8 (PMC9310477; doi:10.1186/s40462-022-00331-8)
Supplement: Supplementary file 3 — Additional file 3. Contains supplementary figures illustrating the soil, bioclimatic, and vegetation covariate layers used in the anthrax suitability modeling. [file 40462_2022_331_MOESM3_ESM.pdf]

Supplementary Figures

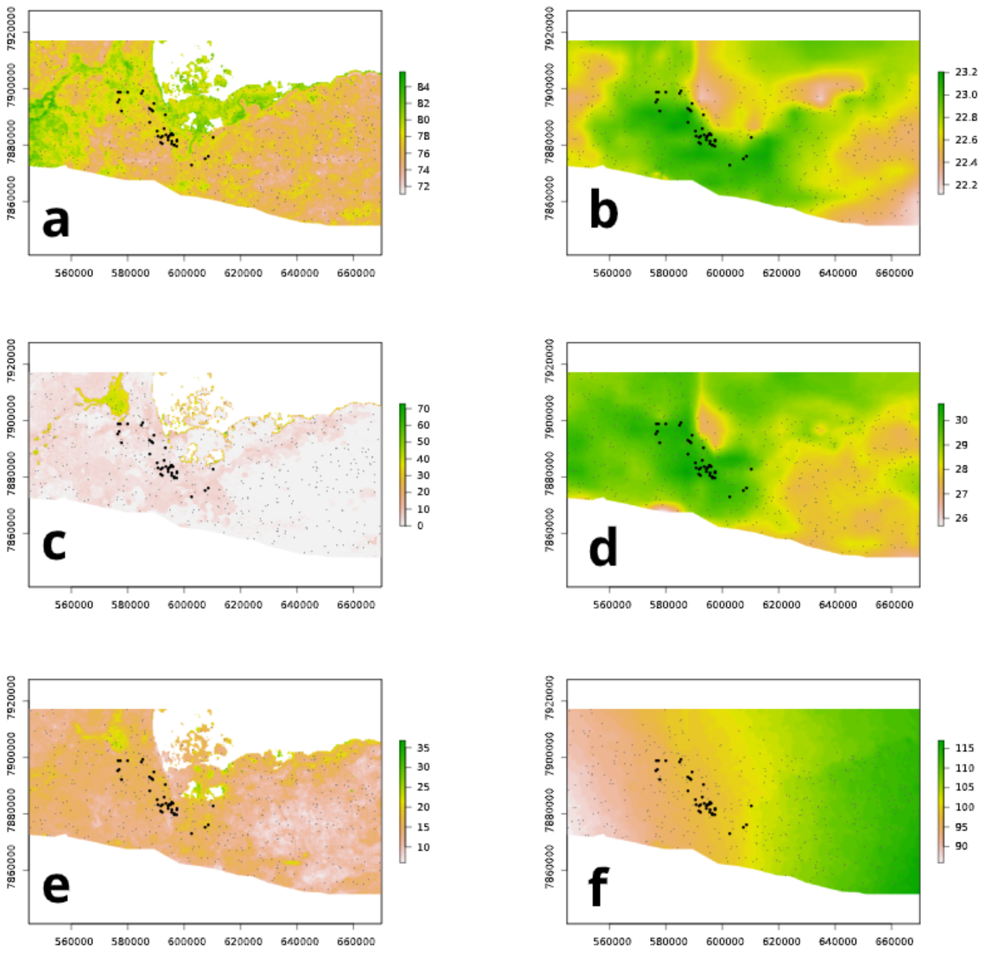

Figure 1 Soil and bioclimatic variables used for the 2009 and 2010 predictive anthrax risk map based on the final MaxEnt model. Larger black points are the locations of the carcasses used as presence locations for the model, whereas smaller gray points are the 422 background locations used to parameterize the model. a) Soil pH in H_2O; b) Mean annual temperature (bio1); c) Soil organic carbon content; d) Mean temperature range (bio7); e) Soil cation exchange capacity; f) Precipitation of the wettest month (bio13).

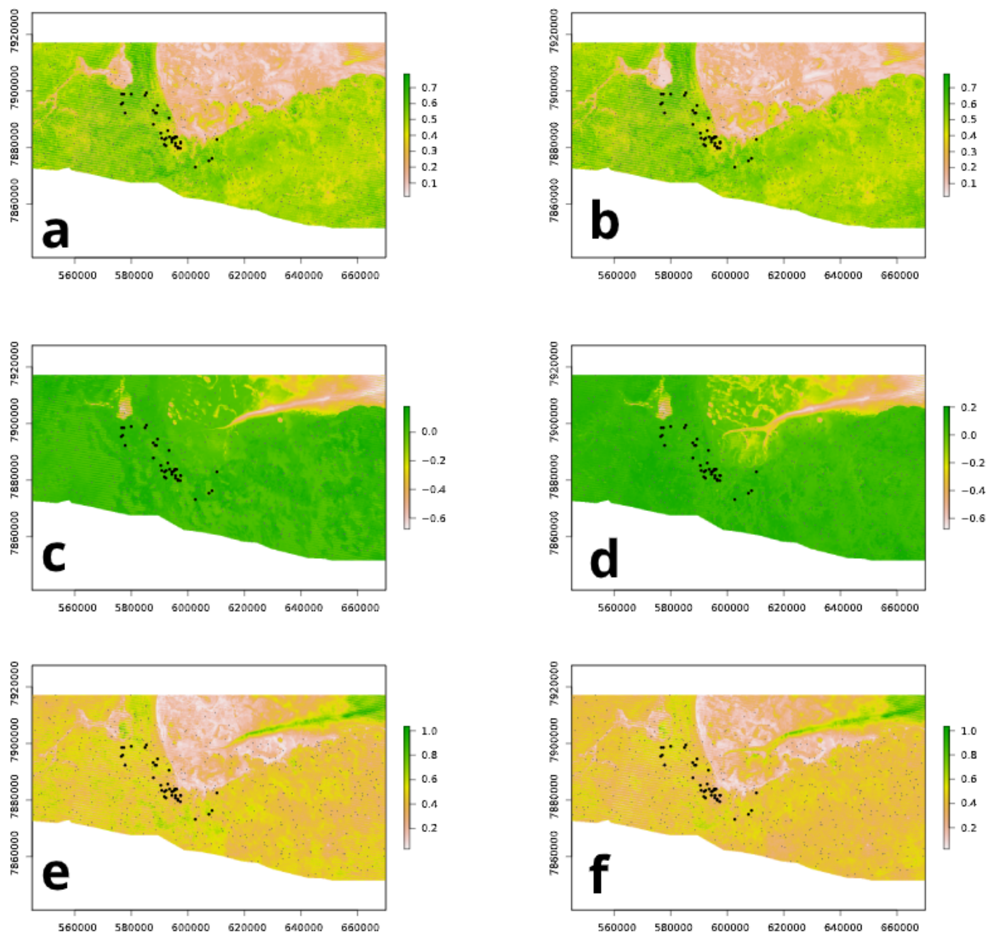

Figure 2 Environmental variables used for the 2009 (left column) and 2010 (right column) predictive anthrax risk map based on the final MaxEnt model. Larger black points are the presence locations and smaller gray points are the randomly generated background sampling points. a) Maximum NDVI 2007-2009; b) Maximum NDVI 2009-2010; c) Minimum NDVI 2007-2009; d) Minimum NDVI 2008-2010; e) Range NDVI 2007-2009; f) Range NDVI 2008-2010.
